# Anticancer role of mango (*Mangifera indica L.*) peel and seed kernel extracts against 7,12- dimethylbenz[a]anthracene-induced mammary carcinogenesis in female rats

**DOI:** 10.1038/s41598-023-34626-6

**Published:** 2023-05-11

**Authors:** Nadia Z. Shaban, Fatma H. El-Rashidy, Amany H. Adam, Doha M. Beltagy, Alaa E. Ali, Ahmed A. Abde-Alaziz, Iman M. Talaat

**Affiliations:** 1 Biochemistry Department, Faculty of Science, Alexandria University, Alexandria, 21511 Egypt; 2 Chemistry Department, Faculty of Science, Damanhour University, Damanhour, Egypt; 3 Endocrinology Unit, Department of Internal Medicine, Faculty of Medicine, Alexandria University, Alexandria, Egypt; 4 Pathology Department, Faculty of Medicine, Alexandria University, Alexandria, Egypt; 5 Clinical Sciences Department, College of Medicine, University of Sharjah, Sharjah, UAE

**Keywords:** Biochemistry, Biotechnology, Cell biology, Drug discovery, Molecular biology, Oncology

## Abstract

Breast cancer is the second leading cause of cancer death among women. The present study is an effort to reveal the antiproliferative and antioxidant actions of mango seed kernel extract (KE), peel extract (**PE**), and their combination (**KEPE**) on mammary tumors induced by 7,12 dimethylbenz[a]anthracene (**DMBA**). Seven groups of adult female Sprague–Dawley rats were prepared, including **C**: (control), **DMBA:** (rats were administered with **DMBA**), (**DMBA-KE),** (**DMBA-PE),** and (**DMBA-KEPE**): rats were administered with **DMBA** and then treated with **KE, PE, and (both KE** and **PE**), respectively, (**KE)** and (**PE)**: rats were administered with **KE** and **PE**, separately. The study focused on the assessment of markers of endocrine derangement [serum 17-β estradiol (E2)], apoptosis [caspase-3 and deoxyribonucleic acid fragmentation (DNAF)], and oxidative stress [lipid peroxidation and antioxidants (glutathione, glutathione-S-transferase, glutathione reductase, glutathione peroxidase, and superoxide dismutase)]. Histopathological examination and immunohistochemical expression of caspase-3 and estrogen receptor-α (ER-α) in mammary gland tissues (MGTs) were determined, as well as the characterization of mango extracts. The results showed that **DMBA** administration induced mammary tumors by increasing cell proliferation and evading apoptosis. In addition, **DMBA** administration caused oxidative stress by the production of reactive oxygen species, which increased lipid peroxidation and decreased cellular antioxidants, allowing cancer to progress. In contrast, treatment with **DMBA-KE, DMBA-PE, or DMBA-KEPE** diminished mammary tumors induced by **DMBA**, where they reduced oxidative stress via increased antioxidant parameters including reduced glutathione, superoxide dismutase, total glutathione peroxidase, glutathione reductase, and glutathione S-transferase. Also, different treatments decreased proliferation through the reduction of E2, and ER-α expression levels. However, these treatments increased the apoptosis of unwanted cells as they increased caspase-3 activity and DNAF. All these changes led to the prevention of breast injuries and the reduction of mammary tumors. This demonstrates that the contents of mango extracts, especially phenolics and flavonoids, have an important role in mammary tumor treatment through their potential antioxidant, antiproliferative, proapoptotic, and anti-estrogenic effects. KE and PE administration for 4 weeks had no adverse effects. **Conclusion:** Each of KE, PE, and KEPE has a therapeutic effect against DMBA-induced mammary tumors via induction of apoptosis and reduction of each of the OS, proliferation, and estrogenic effects. So, they can play an important role in the pharmacological tole.

## Introduction

The breast is the leading cancer site in women around the world. Breast cancer is the major cause of female cancer deaths in nearly all countries, with the exception of the most economically advanced, which is currently second to lung cancer^1^. In our country, Egypt, breast cancer occupies the second rank after liver cancer^2^. The incidence of breast cancer in most countries has been rising for more than 40 years. However, its incidence in a few other countries including North America, Canada, Australia, and France, has been diminishing since 2000–2005. Generally, earlier detection and better treatment account for achievement^3^. Several epidemiological studies have been conducted on the risk factors for breast cancer; it is challenging to formulate an overall assessment, particularly insofar as the identified risk factors interact and differ depending on whether the cancers happen before or after menopause and depending on their histological, biological (receptors), or molecular characteristics^1,2^. Additionally, their frequency changes throughout time and from one area to another. The degree of relative risk for the majority of the factors reaches ≤ 2, while, the risk levels associated with tobacco consumption reach values from 10 to 20, and in some cases even higher. The decision-making process for selecting the most efficient primary prevention measures is facilitated by an estimation of the proportion of risk related to a specific factor (based on the degree of risk and frequency of exposure)^4–6^.

A tumor's carcinogenesis causes aberrant cell growth and death. According to recent research, the development and carcinogenesis of malignant tumors are significantly correlated with the prevention of apoptosis^7^. Apoptosis is an ordered and orchestrated cellular process that occurs in physiological conditions, including growth, development, reproductive age, and normal organ and tissue architecture and function. Additionally, apoptosis has a connection to the development of malignant tumors, the development of proliferative illnesses, and other pathogenic processes^8^. Apoptosis is generally characterized by distinct morphological characteristics and energy-dependent biochemical mechanisms. Morphological hallmarks of apoptosis are chromatin condensation, nuclear fragmentation, membrane blebbing, ultrastructural modification of cytoplasmic organelles, and a loss of membrane integrity^9,10^. Broadly, three main types of biochemical changes can be observed in apoptosis; activation of caspases, DNA and protein breakdown, and membrane changes and recognition by phagocytic cells^9–11^.

Polycyclic aromatic hydrocarbons are present in cigarette smoke, vehicle exhaust, and charcoal-grilled foods^12^. 7,12-Dimethylbenz(a)anthracene (DMBA) is a type of polycyclic aromatic hydrocarbon, that is a lipophilic carcinogen. It has the ability to cause a variety of cancers, including breast cancer, ovarian cancer, leukemia, etc. Through gavage, local application, or subcutaneous injection, several researchers frequently utilize DMBA to create animal models (e.g. rats and mice) of breast cancer or leukemia^13^. The early steps in tumorigenesis involve aryl hydrocarbon receptor up-regulation by cytochrome P450 enzymes, which convert DMBA into a mutagenic epoxide intermediate that readily forms DNA adducts. These adducts are associated with DNA mutations and the malignant transformation that is thought to be involved in polycyclic aromatic hydrocarbon-mediated carcinogenesis^14^. DMBA acts as a potent carcinogen by generating various reactive metabolic intermediates, including 3,4-diol-1,2-epoxide, leading to oxidative stress (OS)^1^. OS is a reflection of an imbalance between the systemic expression of reactive oxygen species and a biological system's ability to quickly detoxify the reactive intermediates or repair the damage that results. When a cell's normal redox state is disturbed, peroxides and free radicals are produced, which harm all of the cell's constituent parts, including proteins, lipids, and DNA, and can have toxic effects. Both base damage and DNA strand breakage are brought on by OS from oxidative metabolism. Reactive oxygen species, such as superoxide anion radical, hydroxyl anion radical, and hydrogen peroxide (H_2_O_2_), are produced and are mostly responsible for indirect base damage. Additionally, in redox signaling, some reactive oxidative species serve as messengers for cells. Thus, OS can result in changes to the regular functioning of cellular signaling pathways^15^.

Several studies have recommended that phytochemical products could be a useful strategy to prevent the deleterious effects of carcinogens and mutagens through the inflection of genes that are related to the control of apoptosis, cell proliferation, cell cycle, signal transduction, transcription regulation, and oncogenesis^11,12^. Mango *(Mangifera indica* L.) is grown naturally or cultivated mainly in tropical and subtropical regions. It has been an important herb in indigenous medical systems for over 4000 years^16^. Peel and mango seeds are discarded as waste and are becoming a source of pollution^17,18^**.** Mango seed kernels and peels are rich in polyphenols with potent antioxidative activity^18^. Different parts of mango, such as stem, bark, leaves, and pulp are known for various biomedical applications, including antioxidative and free radical scavenging, anti-inflammatory, anticancer, immunomodulatory, and hepatoprotective properties^19–22^. Mango peel and seed kernels are considered economic sources. The current study is the first report about the evaluation of the therapeutic roles of peel extract (PE), seed kernel extract (KE), and their combination (KEPE) on the mammary tumor in adult female (Sprague–Dawley) rats induced by DMBA. The study focused on the assessment of markers of endocrine derangement [serum 17-β estradiol (E2)], apoptosis [caspase-3 and DNA fragmentation (DNAF)], and OS [lipid peroxidation, and antioxidants (reduced glutathione (GSH), glutathione S-transferase (GST), glutathione reductase (GSR), total glutathione peroxidase (t-GPx), and superoxide dismutase (SOD)] in mammary gland tissues. Also, the histopathological examination of mammary tissues and immunohistochemical expression of caspase-3 and estrogen receptor-α (ER-α) in mammary gland tissues were determined. Also, the effect of these extracts on healthy rats was studied in order to evaluate their toxicity. In addition, the characterizations of mango extracts were assessed.

## Results

### Characterization of PE and KE

#### Total phenolic contents

**Figure 1:**
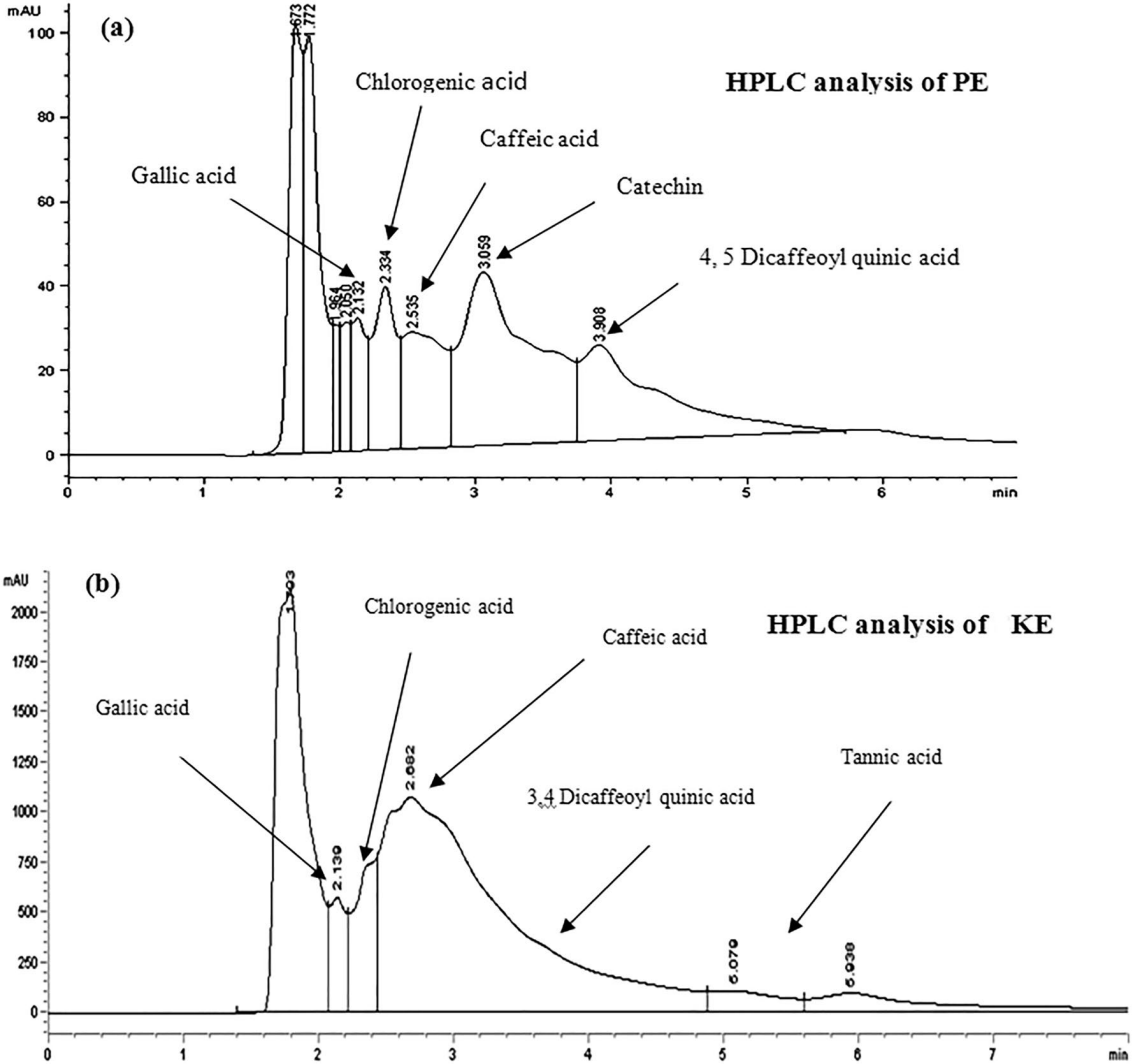
HPLC analysis of polyphenolic compounds in PE and KE (**a** & **b**).

The results showed that total phenolic content in PE and KE are 5293.80 ± 0.00 and 11,227.20 ± 0.00 as mg gallic acid equivalent /100 g of dry mass), respectively. HPLC analysis of polyphenolic compounds in PE and KE and their concentrations are listed in Fig. 1a,b and Table 1.

**Table 1:** HPLC analysis of polyphenolic compounds in PE and KE and their concentrations.

HPLC Products	Concentration (mg/100 g of PE dry mass)	Concentration (mg/100 g of KE dry mass)
Gallic acid	1.5870	20.062
Chlorogenic acid	2.2287	35.928
Caffeic acid	4.8875	1118.2
3,4 Dicaffeoyl quinic acid	–	0.6015
4,5 Dicaffeoyl quinic acid	9.5217	–
Catechin	66.087	0.0043
Tannic acid	–	9.6061

#### Total flavonoid content

The data showed that PE and KE contain 82.11 ± 0.00 and 207.12 ± 0.00 as mg quercetin equivalents /100 g of dry mass), respectively.

#### Total antioxidant capacity

The results revealed that the total antioxidant capacities of PE and KE are 88.230 ± 0.001 and 280.800 ± 0.001 as mg ascorbic acid equivalents /100 g of dry mass, respectively.

### Total protein level in mammary gland tissues (MGTs) of different studied groups

Total protein level in rats administered with DMBA was non-significantly decreased by about 0.2% compared to the C group (Table 2). Rats treated with KE and PE after DMBA administration [(DMBA-KE) and (DMBA-PE) groups] showed a nonsignificant decrease in total protein level by about 7.3% and 0.42%, respectively, compared to the DMBA group. Treatment with both KE and PE after DMBA administration (DMBA-KEPE) group increased total protein level non-significantly by about 3.8% compared to the DMBA group. Administration of KE or PE separately increased total protein level non-significantly by about 2.78% and 1.1%, respectively, compared to the C group.

**Table 2:** Effect of different extracts on total protein level in mammary gland tissue and serum E2 in different studied groups.

Studied groups	Status of E2 (Pg/ml)	TP level (mg/g tissue)
C	45.70 ± 1.59	1.187 ± 0.09
DMBA	59.02 ± 0.59^a^	1.185 ± 0.07
DMBA-KE	27.417 ± 1.63^ab^	1.099 ± 0.068
DMBA-PE	43.17 ± 0.71^bc^	1.19 ± 0.14
DMBA-KEPE	38.19 ± 0.703^abcd^	1.23 ± 0.16
KE	21.86 ± 1.62^abcde^	1.22 ± 0.05
PE	42.14 ± 1.77^bcef^	1.20 ± 0.22

The values are expressed as means ± S.D. for seven rats. Within each row, the values with different letters are significantly different at *p* ≤ 0.05 (a: Significant with C group; b: Significant with DMBA group; c: Significant with DMBA-KE group; d: Significant with DMBA-PE group; e: Significant with DMBA-KEPE group; f: Significant with KE group). Group C: control rats; group (DMBA): rats were administered orally with (80 mg/kg) of DMBA as a single dose; group (DMBA-KE): rats treated with KE for 4 weeks after the 9th week of DMBA administration; group (DMBA-PE): rats treated with PE for 4 weeks after the 9th week of DMBA administration; group (DMBA-KEPE): rats treated with both KE and mango PE for 4 weeks after the 9th week of DMBA administration; group (KE): rats treated with KE only for 4 weeks; group (PE): rats treated with PE only for 4 weeks. Where E2 = 17-β estradiol and TP = total protein.

### Total body weight and weight of right and left mammary gland tissues of different studied groups

**Figure 2:**
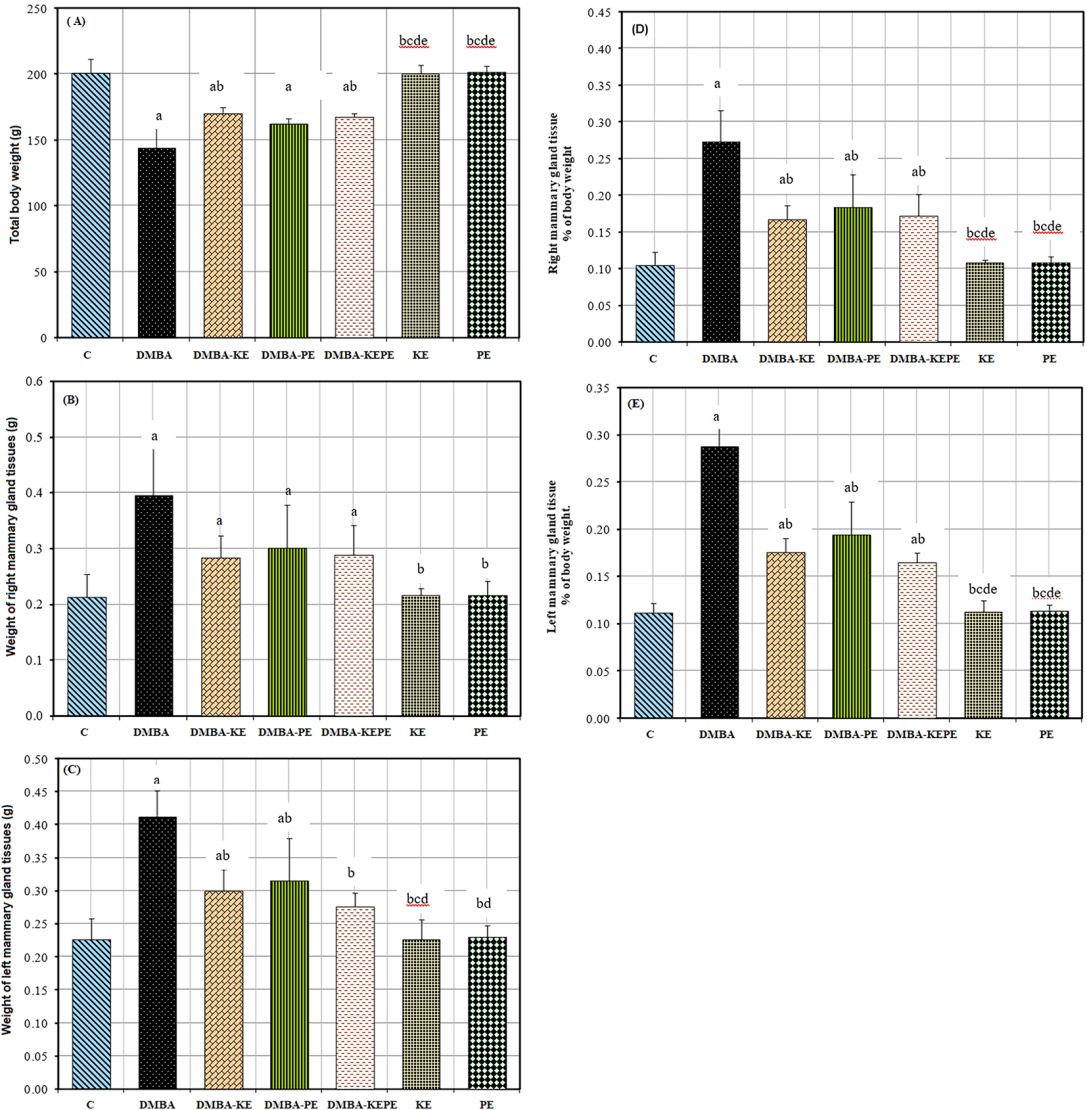
Effect of 7, 12- Dimethyl-benz[a]anthracene (DMBA), KE, PE, and (KEPE) on the total body weight and weight of mammary glands. (**A**): total body weight; (**B**): weight of right MGT and (C): weight of left MGT. Where group C-control: rats were administered orally with a single dose of 4 ml sesame oil/kg bm; group (DMBA): rats were administered orally with (80 mg/kg) of DMBA as a single dose; group (DMBA-KE): rats treated with KE for 4 weeks after the 9th week of DMBA administration; group (DMBA-PE): rats treated with PE for 4 weeks after the 9th week of DMBA administration; group (DMBA-KEPE): rats treated with both KE and PE for 4 weeks after the 9th week of DMBA administration; group (KE): rats treated with KE only for 4 weeks; group (PE): rats treated with PE only for 4 weeks. Results are given as means ± S. D. for seven rats. Values with different letters are significantly different at *p* ≤ 0.05 (a: Significant with C group; b: Significant with DMBA group; c: Significant with DMBA-KE group; d: Significant with DMBA-PE group; e: Significant with DMBA-KEPE group; f: Significant with KE group).

Figure 2A shows that the body weight of rats after DMBA administration was significantly decreased as compared to the C group. However, treatment with KE, PE, and KEPE after DMBA administration improved the body weight of rats. Administration of KE and PE to healthy rats, separately, had no effect on body weight. Otherwise, the weights of the right and left MGTs in rats administered with DMBA were increased as compared to the C group (Fig. 2B,C). While their weights were decreased in rats treated with KE, PE, and KEPE after DMBA administration. Also, the administration of KE and PE separately had no effect on the weight of the right and left MGTs (Fig. 2B,C). DMBA administration caused a significant elevation in the right and left MGTs as % of body weight as compared to the C group (Fig. 2D,E). While treatment with KE, BE, or KEPE after DMBA administration significantly decreased the right and left MGTs as % of body weight when compared to the DMBA group. Administration of KE or PE alone caused a non-significant increase in right and left MGTs as % of body weight as compared to the C group (Fig. 2C,D).

### Caspase-3 activities

**Figure 3:**
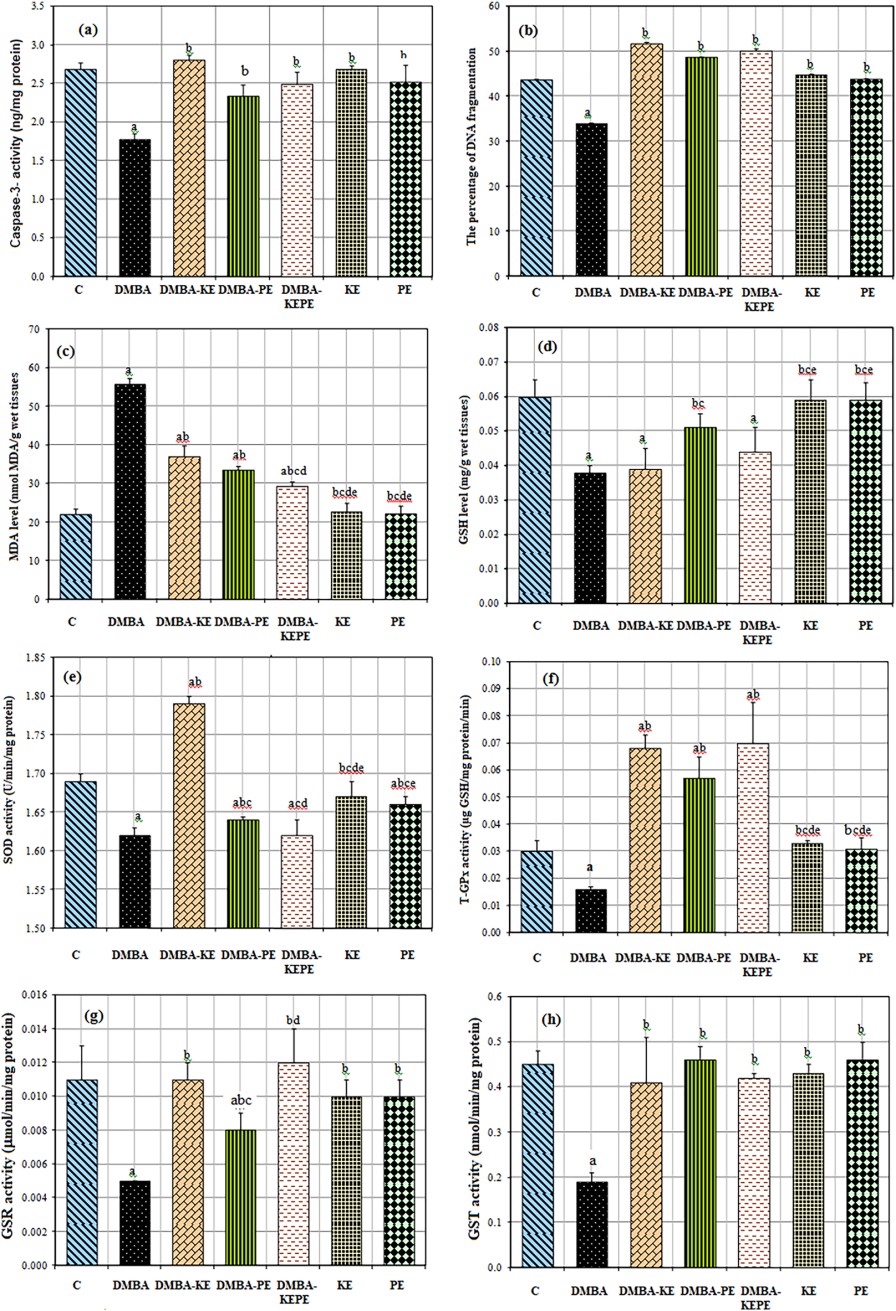
Effect of 7, 12- Dimethyl-benz[a]anthracene (D), KE, PE, and (KEPE) on the markers of apoptosis and oxidative stress. Caspase-3 activity (**a**); percentage of DNA fragmentation (**b**); MDA (**c**); GSH (**d**); SOD activity (**e**); t-GPx activity (**f**); GSR activity (**g**) and GST activity (**h**) in rat mammary gland tissues. Where group C-control: rats were administered orally with a single dose of 4 ml sesame oil/kg bm; group (DMBA): rats were administered orally with (80 mg/kg) of DMBA as a single dose; group (DMBA-KE): rats treated with KE for 4 weeks after the 9th week of DMBA administration; group (DMBA-PE): rats treated with PE for 4 weeks after the 9th week of DMBA administration; group (DMBA-KEPE): rats treated with both KE and PE for 4 weeks after the 9th week of DMBA administration; group (KE): rats treated with KE only for 4 weeks; group (PE): rats treated with PE only for 4 weeks. Results are given as means ± S.D. for seven rats. Values with different letters are significantly different at *p* ≤ 0.05 (a: Significant with C group; b: Significant with DMBA group; c: Significant with DMBA-KE group; d: Significant with DMBA-PE group; e: Significant with DMBA-KEPE group; f: Significant with KE group).

Caspase-3 activity in the DMBA group was significantly decreased by about 33.6% compared to the C group (Fig. 3a). However, its activity in (DMBA-KE), (DMBA-PE) and (DMBA-KEPE) groups was increased by about 57.3%, 31.5%, and 40%, respectively, compared to the DMBA group. PE administration decreased caspase-3 activity nonsignificantly by about 6% as compared to the C group, while KE had no effect.

### Percentage of DNAF

The DNAF level in DMBA was significantly decreased by about 22.4% compared to the C group (Fig. 3b). However, its levels in the (DMBA-KE), (DMBA-PE), and (DMBA-KEPE) groups were increased significantly by about 52.8%, 43.6%, and 47.9%, respectively, as compared to the DMBA group. Administration of KE or PE, separately, increased DNAF non-significantly by about 2.5% and 0.55%, respectively, compared to the C group.

### Serum E2 level

Administration of DMBA significantly increased serum E_2_ level by about 29% compared to the C group (Table 2). In contrast, E_2_ levels in the (DMBA-KE), (DMBA-PE), and (DMBA-KEPE) groups were significantly decreased by about 53.5%, 27%, and 35.3%, respectively, compared to the DMBA group. E2 level was decreased in the KE group (significantly by about 52.2%) and in the PE group (non-significantly by about 8%) as compared to the C group.

### Malondialdehyde (MDA) level

DMBA administration extremely significantly increased the MDA level by about 154% compared to the C group (Fig. 3c). Otherwise, MDA levels in the (DMBA-KE), (DMBA-PE), and (DMBA-KEPE) groups were significantly decreased by about 33.5%, 39.6%, and 47.4%, respectively, as compared to the DMBA group. Administration of KE and PE separately non-significantly increased MDA levels by about 3.65% and 1%, respectively, compared to the C group.

### GSH level

DMBA administration significantly decreased GSH level by about 37% compared to the C group (Fig. 3d). GSH levels in the (DMBA-KE), (DMBA-PE) and (DMBA-KEPE) groups were increased by about 3% (non-significantly), 34% (significantly), and 16% (non-significantly), respectively, compared to the DMBA group. Administration of KE and PE, separately, nonsignificantly decreased GSH levels by about 1.7% and 1.7%, respectively, compared to the C group.

### SOD activity

Administration of DMBA significantly decreased SOD activity by about 4% compared to the C group (Fig. 3e). SOD activities in (DMBA-KE) and (DMBA-PE) groups were significantly increased by about 10.5% and 1.24%, respectively, compared to the DMBA group. However, SOD activity in the (DMBA-KEPE) group did not change as compared to the DMBA group. Administration of KE or PE, separately, decreased SOD activity by about 1.2% (non-significant) and 1.78% (significant), respectively, as compared to the C group.

### T-GPx activity

DMBA administration non-significantly decreased t-GPx activity by about 46.7% compared to the C group (Fig. 3f). In contrast, t-GPx activities in (DMBA-KE), (DMBA-PE), and (DMBA-KEPE) groups were significantly increased by about 325%, 256%, and 337.3%, respectively, compared to the DMBA group. Administration of KE or PE, separately, non-significantly increased t-GPx activity by about 10% and 3.3%, respectively, as compared to the C group.

### GSR activity

Administration of DMBA highly significantly decreased GSR activity by about 54.5% compared to the C group (Fig. 3g). Rats treated with KE and PE after DMBA administration in DMBA-KE and DMBA-PE groups showed a highly significant increase in GSR activity by about 120% and 60%, respectively, compared to the DMBA group. Treatment with both KE and PE after DMBA administration in the DMBA-KEPE group extremely significantly increased GSR activity by about 140% compared to the DMBA group. Administration of KE or PE, separately, decreased GSR activity non-significantly by about 9% and 2.2%, respectively, compared to the C group.

### GST activity

DMBA administration highly significantly decreased GST activity by about 57.8% compared to the C group (Fig. 3h). GST activities in (DMBA-KE), (DMBA-PE), and (DMBA-KEPE) groups significantly increased by about 115.8%, 142%, and 121%, respectively, compared to the DMBA group. GST activity was decreased non-significantly after KE administration by about 4.4%, while its activity increased non-significantly after PE administration by about 2.2% as compared to the C group.

### Histopathological and Immunohistochemical examination

**Figure 4:**
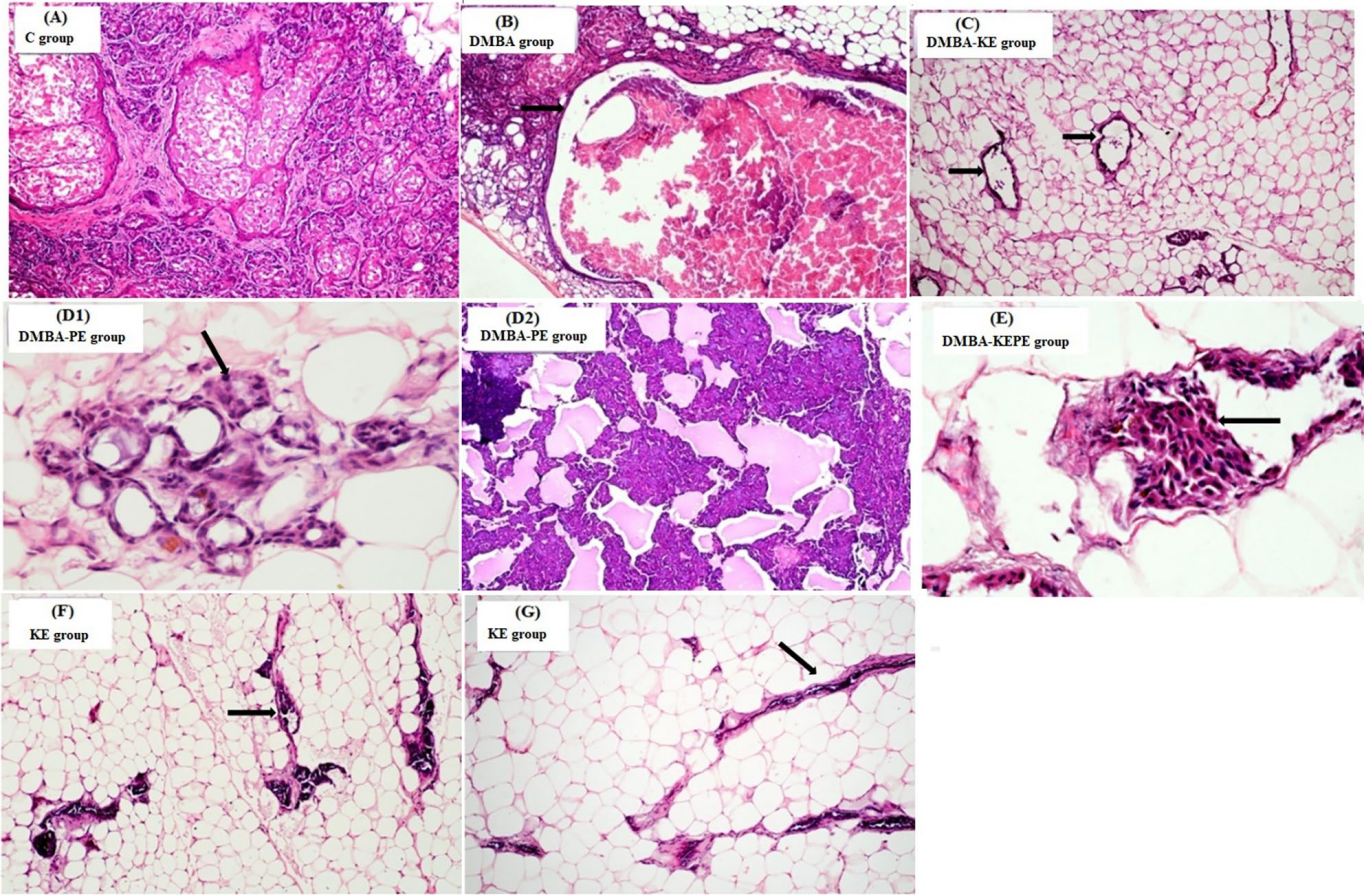
Histopathological examination of mammary gland tissues in different studied groups. The C group (**A**) shows normal mammary gland tissue. DMBA group (**B**) shows dilated duct containing inspissated secretions (arrow) and atypical epithelial cells. DMBA-KE group (**C**) reveals proliferated dilated breast ducts (arrows) embedded into fat tissue with no residual tumor tissue. DMBA-PE group (**D1**) shows lobular architecture with a ductal carcinoma focus (arrow), and (**D2**) reveals lymph nodes showing reactive changes. DMBA-KEPE group (**E**) revealed breast tissue with a focus showing atypical cells (arrow). KE group (**F**) and PE group (**G**) show normal branched breast ducts within fat tissue (arrows). Figure 4A,B,C,D2,F,G: H & E stain × 100. Figure 4D1,E: H & E stain × 400.

Examination of the C group revealed normal MGTs composed of lobules and dilated ducts. The lobules are lined by cuboidal cells and an outer layer of myoepithelial cells, and the stroma in between is formed of fibrous tissue. (Fig. 4A). DMBA administration revealed MGTs with a dilated duct containing inspissated secretions. A neoplastic growth is seen to consist of atypical epithelial cells, pleomorphic with hyperchromatic, overlapped nuclei (Fig. 4B). Adenocarcinoma, which is characterized by sheets of tumor cells separated by small cystic spaces, is found. Highly invasive tumor cells are seen in the adjacent stroma. Treatment with KE after DMBA administration revealed proliferated dilated breast ducts embedded into the surrounding fat tissue with no residual tumor tissue that could be identified (Fig. 4C). Treatment with PE after DMBA administration revealed lobular architecture with a focus on ductal carcinoma where the cells are mildly pleomorphic, and hyperchromatic with increased nucleocytoplasmic ratio (Fig. 4D1,D2). Treatment with KE and PE together after DMBA administration revealed MGTs with a focus showing atypical cells (Fig. 4E). Administration with KE only showed normal branched breast ducts with the normal distribution of fat tissue (Fig. 4F), whereas administration of PE showed normal breast ducts and normal distribution of adipocytes (Fig. 4G).

**Figure 5:**
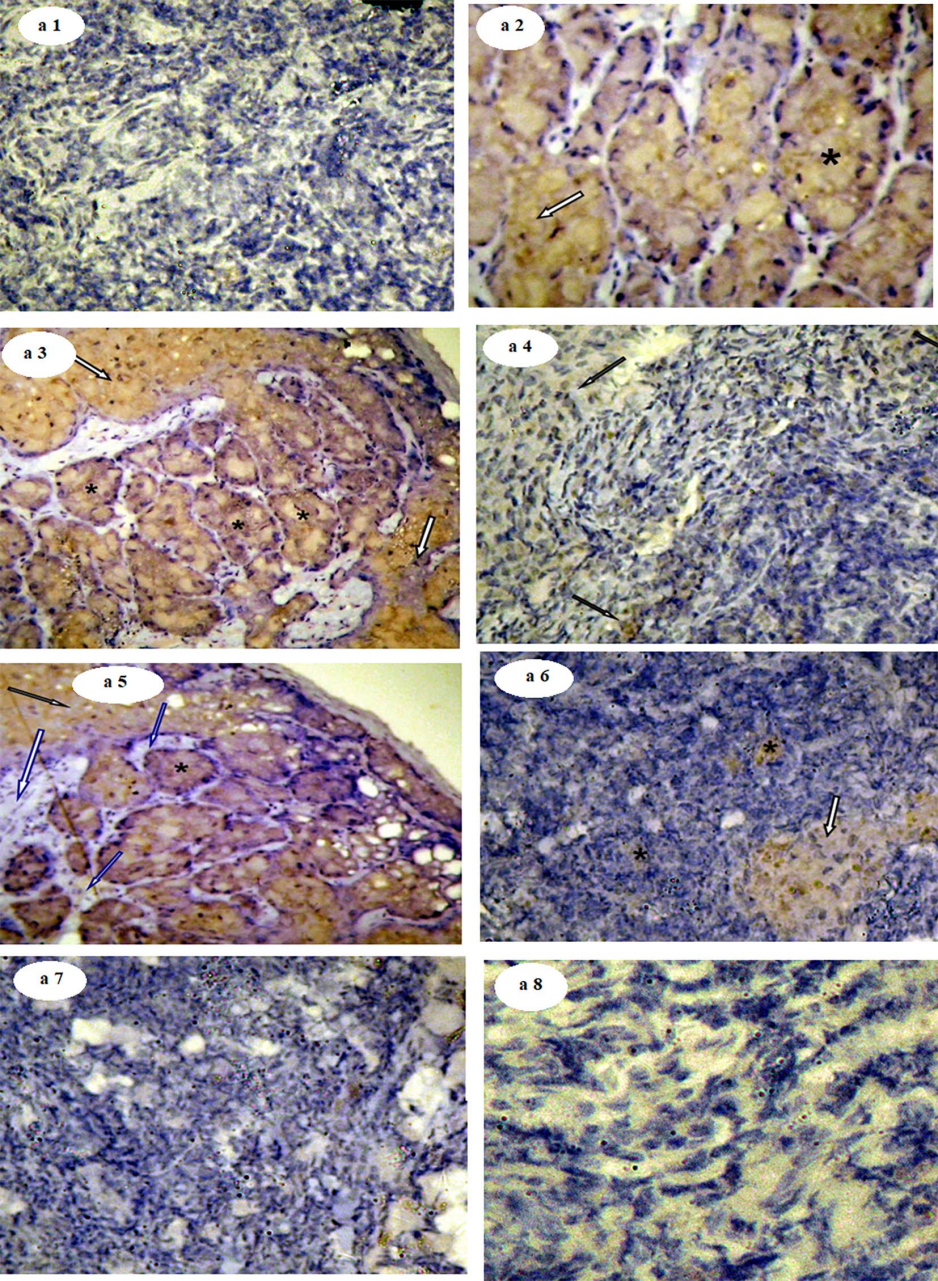
Photomicrographs of ER-ir in mammary gland tissues of different studied groups. The immunohistochemical expression of ER-α in MGTs sections showed a varying degree of positive and negative reactions for ER-α (Fig. 5a1–a8).

Otherwise, the immunohistochemical expression of ER-α in MGTs sections of different studied groups showed a varying degree of positive and negative reactions for ER-α (Fig. 5a1–a8). The control group (a1) showed a very low reaction for ER. MGTs sections from the DMBA group (a2 and a3) revealed a highly positive reaction for ER with the characteristic cytoplasm brown staining around the ducts (arrows) and many metastatic foci formed inside the adipocytes (stars). DMBA-KE group (a4) showed very few positive reactions for ER indicated by the brown staining of the cytoplasm. This section is very similar to the normal. DMBA-PE group (a5) showed a relatively moderate positive reaction for ER indicated by the brown staining of the cytoplasm (arrow) and metastatic foci (star) with many areas appearing to be treated showing normal sections (blue arrows). DMBA-KEPE group (a6) showed a mild positive reaction for ER indicated by the brown staining of the cytoplasm (arrow) and tiny metastatic foci (star). MGTs sections of KE (a7) and PE (a8) groups showed very low reaction for ER as the normal section.

**Figure 6:**
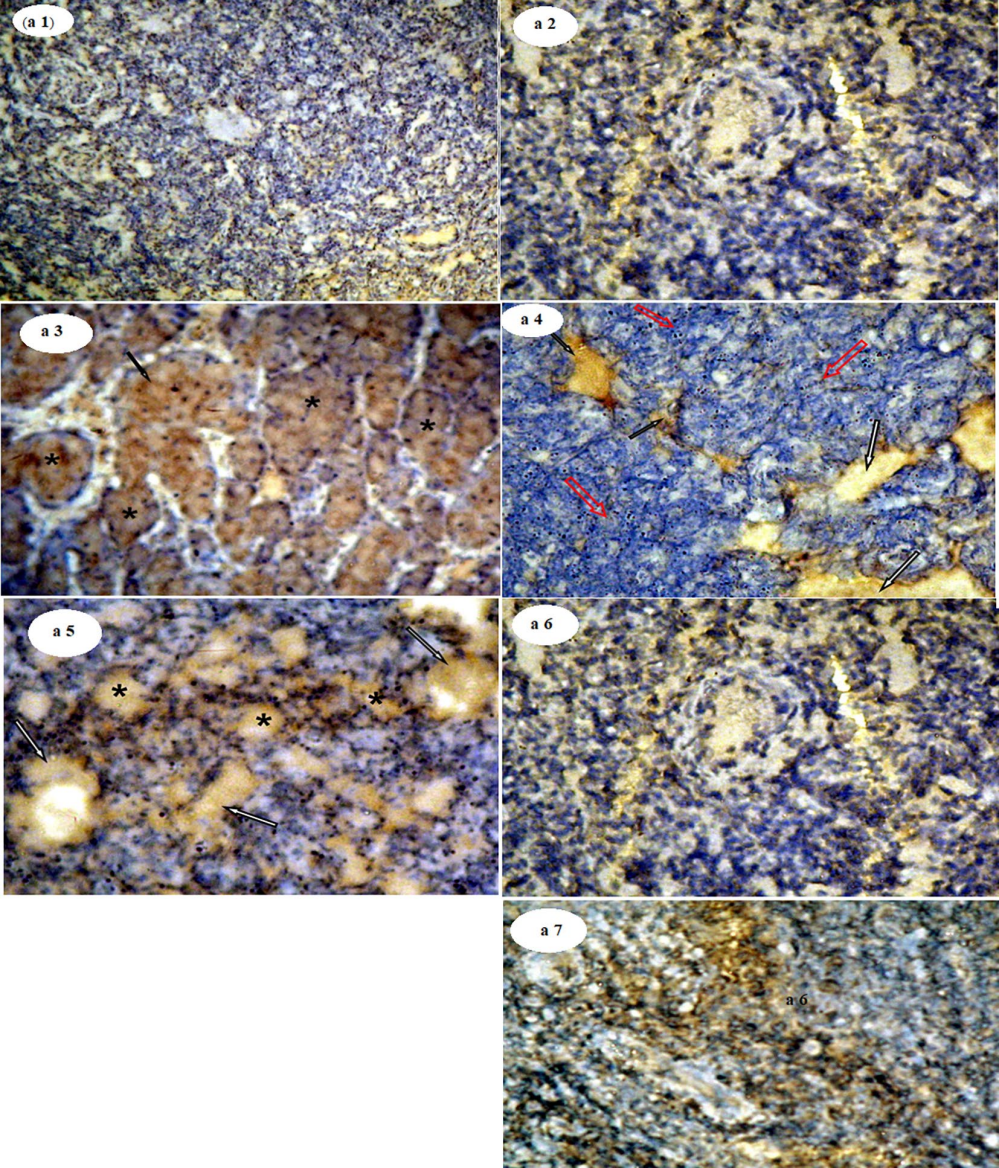
Photomicrographs of Caspase-3-ir in mammary gland tissues of different studied groups. The immunohistochemical expression of caspase-3 in mammary gland tissue sections revealed a varying degree of positive and negative reactions for caspase-3 (Fig. 6a1–a7).

Also, Fig. 6a1–a7 revealed the photomicrographs of caspase-3-ir in MGTs sections of different studied groups. The control group (a1) showed a very low reaction for caspase-3. MGTs sections from the DMBA group (a2) show a negative reaction for caspase-3. DMBA-KE group (a3) showed a strong positive reaction of caspase-3 with the characteristic cytoplasm brown staining around the ducts (arrow) and many metastatic foci formed inside adipocytes (star). DMBA-PE group (a4) showed a moderate positive reaction for caspase-3 indicated by the brown staining of the cytoplasm and metastatic foci (arrow). DMBA-KEPE group (a5) showed a mild positive reaction for caspase-3 indicated by the brown staining of the cytoplasm (arrow) and tiny metastatic foci (star). MGTs sections of KE (a6) and PE (a7) groups showed a relatively normal reaction for caspase-3 as the normal section.

**Figure 7:**
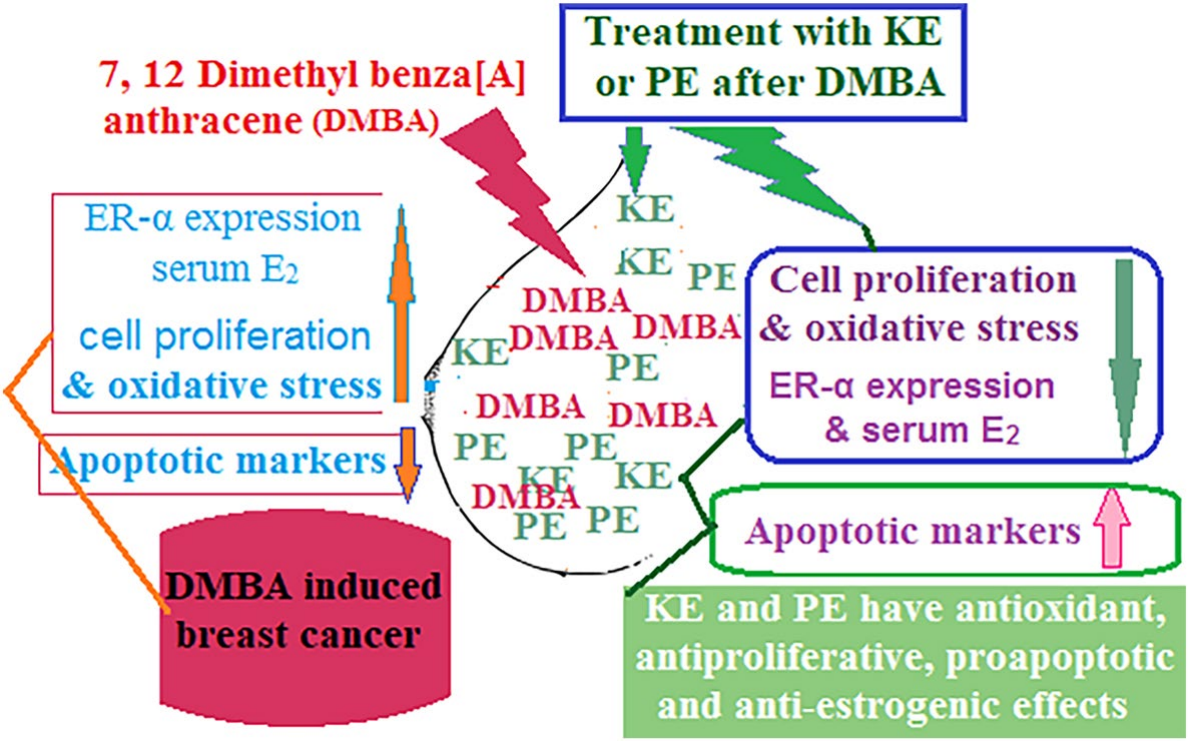
Presents the research's conclusions as a whole.

**An overview of the results:** Figure 7 presents the research's conclusions.

## Discussion

Rat mammary tumors induced by the administration of DMBA are morphologically and histologically like human mammary tumors. Therefore, they have been employed to investigate the chemopreventive potential of medicinal plants and dietary agents^1^. The health benefits attributed to fruit and vegetable consumption are due to many factors, including vitamins, minerals, dietary fiber, and phytochemical content. Mango (*Mangifera indica* L.) has been the focus of attention of many researchers searching for potent antioxidants. Different parts of the mango, such as the stem, bark, leaves, and pulp, are known for various biomedical applications, including antioxidative and free radical scavenging, anti-inflammatory, and anticancer activities^22^. As shown from our data, the antioxidant capacities of KE and PE are very high, and this may be due to their contents, especially phenolics, and flavonoids, which are present in large amounts, and their variation. Therefore, the current study was designed to reveal the antioxidant, antiproliferative, and antitumor effects of PE, KE, and their combination (KEPE) on the female rat mammary tumor induced by DMBA.

The histopathological examination of the mammary glands of female rats after DMBA administration showed that DMBA induced breast cancer. Also, in the DMBA group, we noticed that there was a significant reduction in final total body weight with significant elevations in the right and left MGTs weights as a percentage of body weight when compared to the C group. All these observations confirmed that DMBA caused breast cancer.

Otherwise, the biochemical data confirmed the histological results. The biochemical outcomes showed a significant elevation in MDA level with significant declines in GSH level and the activities of GSR, SOD, t-GPx, and GST in MGTs in the DMBA group. This indicates that DMBA caused OS and this may be due to the toxic effects of DMBA and its metabolites, including epoxides such as D-3,4-dihydrodiol-1,2-epoxide, quinines, and oxyradicals. These free radicals increased the lipid peroxidation of polyunsaturated fatty acids in membranes, caused a loss of cell integrity, increased membrane permeability, and altered both calcium homeostasis and inner membrane potential, resulting in cell death^23–27^. GSH plays a regulatory role in protecting cells against cytotoxic and carcinogenic chemicals since it acts as a nucleophilic scavenger of several compounds through chemical and enzymatic mechanisms as well as acting as a cofactor in GPx-mediated obliteration of H_2_O_2_^25,26^**.** Diminution of GSH after DMBA administration may be owed to increase its need for the metabolism of lipid hydroperoxide by GPx. Also, the decline in GSH level may be due to its interaction with any free radicals, including 3,4-diol-1,2-epoxide^24,28,29^. Also, the reduction in GSR activity, as shown by our results, led to a decline in GSH level. The changes in the rate of cancer cell proliferation are accompanied by changes in their intracellular GSH levels, and consequently, this could be reflected in their antioxidant machinery^10^. Moreover, the reduction in GSH level may be related to the induction of a mammary tumor by DMBA that led to a significant block in gut GSH release^30^.

GSR plays a key role in cellular defense against OS by preventing the accumulation of GSSG and thus maintaining the redox state. GSR is also important in the synthesis of DNA precursors and proton transport across membranes^25,26,31^. It is used as a tumor marker in certain cancers, such as breast and oral cancer^32^. The decline in GSR activity after DMBA administration is probably due to its inhibition by DMBA and/or its metabolites. SOD is the original line of defense in the body against superoxide anion radicals and is considered the mainly efficient antioxidant^33–35^. Our results have shown that the specific activity of SOD was reduced in the DMBA group, indicating its inhibition by DMBA or its metabolites^36^. Also, inhibition of SOD may be due to the oxidation of its cysteine residue by a superoxide anion radical and/or H2O2 resulting from lipid peroxidation^32^. Furthermore, the inhibition of t-GPx activity led to the accumulation of H2O2, resulting in SOD inhibition. The GPx supplies the second line of defense against hydroperoxides by catalyzing the reduction of H2O2 and other organic hydroperoxides (ROOH) in the presence of GSH. The decline in t-GPx activity in DMBA group may be related to the reduction of GSH, besides its inhibition by DMBA and its reactive metabolites^9^. Otherwise, GST is considered a drug-metabolizing enzyme and is GSH-dependent^37^, so the decrease in its activity in the DMBA group may be due to a decrease in GSH level^32^.

In addition, the present results have shown that the apoptotic markers (DNAF and caspase-3 activity) were significantly decreased in the DMBA group. And this may be owing to the overexpression of caspase-3 inhibitors and their survival in tumor cells. The reduction in apoptotic markers may reflect the down-regulation of death receptors (cell-surface receptors and death domains) and/or mitochondrial pathways (Bcl-2 family of proteins and cytochrome c) of apoptosis^38^. Otherwise, active metabolites of DMBA covalently bind to DNA, forming stable adducts that lead to the induction of mutations in critical genes and subsequently to the neoplastic transformation of the target cells^9^. Also, our results revealed a very low immunohistochemical expression of caspase-3 in the DMBA group, since this result was confirmed by the reduction in caspase-3 activity.

On the other hand, the significant elevation in serum E2 level in the DMBA group indicates that there is a relation between serum E2 level and mammary carcinogenesis. The elevation of the E2 level stimulates the generation of free radicals such as superoxide anion radicals, H2O2, and quinine. In addition, E2 in breast tissue is transformed to catechol estrogen-3,4-quinone, which reacts with adenine and guanine in DNA, forming unstable adducts that can lead to breast carcinoma^39,40^. On the other hand, the significant elevation in the expression of ER-α in the DMBA group indicates that the carcinogenic effects of estrogen may also be mediated in an estrogen receptor-dependent manner (estrogen activity via the estrogen receptor). Thus, based on these results, DMBA induces mammary carcinogenesis by increasing cell proliferation, suppressing apoptosis, and stimulating the production of free radicals, which induce OS.

On the other hand, the results of the present study showed that the total flavonoid and phenolic contents of KE are about 0.21 g as quercetin equivalents, and 11.23 g as gallic acid equivalents per 100 g of the dried kernel, respectively^17^. Also, the results showed that the total flavonoid and phenolic contents of PE are about 0.08 g as quercetin equivalents and 5.30 g as gallic acid equivalents per 100 g of dried peel. Additionally, the results of the HPLC analysis of PE showed the incidence of chlorogenic acid, 3,4-dicaffeoylquinic acid, gallic acid, caffeic acid, and catechin. Also, previous studies demonstrated that PE contains protocatechuic acid, P-coumaric acid, ellagic acid, mangiferin, quercetin, rhamnetin, kaempferol, and their related conjugates^23^. These conjugates include mangiferin gallate, isomangiferin, isomangiferin gallate, quercetin 3-O-galactoside, quercetin 3-O-glucoside, quercetin 3-O-xyloside, quercetin 3-O-arabinopyranoside, quercetin 3-O-arabinofuranoside, kaempferol 3-O-glucoside, and rhamnetin 3-O-galactoside/glucoside^18^. In addition, PE contains considerable amounts of carotenoids, vitamin C, vitamin E, and anthocyanins^19^. All these compounds in PE and KE have antioxidant, anti-inflammatory, and anticancer activities^19,22^. Consequently, our results showed that KE and PE have high antioxidant capacities, which are equal to 280.8 and 88.23 mg of ascorbic acid equivalent per 100 g of dried kernel and peel, respectively. Our results agree with previous studies, which showed that the antioxidant activity of many fruits such as mango, pomegranate, and *carica papaya* linn is related to phenolics, flavonoids, carotenoids, and vitamins E and C^1,9,10,15^. Where, the antioxidant activity of phenolic and flavonoid compounds may be due to the reactivity of the phenol moiety and their ability to scavenge free radicals, such as DPPH radicals, hydroxyl radicals, and alkyl radicals, via hydrogen or electron donation^24–26^. Therefore, treatment of rats with PE, KE, or KEPE showed attenuation of mammary carcinogenesis induced by DMBA, as shown in all results. The histopathological results showed different morphologies of tumor cells since the tissues revealed mild ductal proliferation. Also, the final total body weight of rats was significantly increased, while the weight of the right MGTs and left MGTs were decreased. So, the MGTs percentage of body weight was decreased, when compared to the DMBA group. This may be related to the protective effect of various contents of PE, KE, and KEPE, especially phenolics and flavonoids, including chlorogenic acid, 3,4-dicaffeoylquinic acid, gallic acid, caffeic acid, catechin, protocatechuic acid, P-coumaric acid, ellagic acid, mangiferin, quercetin, rhamnetin, kaempferol, and their related conjugates. Previous studies revealed that these compounds have antioxidant, antiproliferative, proapoptotic, and anti-estrogenic properties^1,13,14,21,26^. Also, our results confirmed the previous results, where KE, PE, and KEPE revealed that they have antiproliferative, proapoptotic, anti-estrogenic, and antioxidant properties, as discussed below.

The present results demonstrated that treatment of rats with PE, KE, or KEPE after DMBA administration decreased ER-α expression and serum E2 level as compared with the DMBA group. The reduction in serum E2 level may be related to the effect of the contents of these extracts, including phytosterols, phytoestrogens, and different phenolic and flavonoid compounds. These compounds may block receptor function or drastically reduce the level of endogenous estrogen through inhibition of its biosynthesis. Previous studies revealed that phytosterols present in the KE play a potential role in attenuating steroid biosynthesis through the reduction of serum cholesterol levels^41–46^ and downregulating aromatase expression. Also, flavonoids, such as quercetin, inhibit aromatase^47^. Furthermore, flavonoids and phytoestrogens decrease or prevent cell proliferation since they act as agonists of ER-β-dependent pro-apoptotic signaling and antagonists of ER-α which elicit rapid responses. Also, quercetin and naringenin impair ER-α mediated rapid activation of signaling kinases [Extracellular signal-regulated kinase/mitogen-activated protein kinase) and phosphatidylinositol 3-kinase/Protein kinase B)] and cyclin D1 transcription (Cyclin D1 is a protein required for progression), both important for cell proliferation. In addition, they increase apoptosis because they act as E2 mimetics in the presence of ER-β rapidly activating the rapid phosphorylation of protein P38/mitogen-activated kinase and, in turn, the induction of a proapoptotic cascade (as caspase-3 activation) and driving cells to apoptosis. In contrast, they spare normal cells by several mechanisms, which may include the decrease of ROS, regulation of heat shock protein expression, modulation of the signaling pathway, and the release of cytochrome c with subsequent activation of caspase-9 and caspase-3^48^. Otherwise, ellagic acid (as a component of mango extracts) is a natural selective ER-α and ER-β ligand, exhibiting selective estrogen receptor modulator properties ^49^. Furthermore, treatment with PE, KE, or KEPE after DMBA administration suppressed the proliferation of breast cancer by activation of the apoptotic pathways (as shown by the elevation of caspase-3 activities, DNAF, and caspase-3 expression) and consequently reduced the proportion of transformed mammary cancer cells. This indicates that the contents of these extracts, which were mentioned before, played an important role in the induction of apoptosis. Our results agree with previous results^18^, which revealed that phenolic acids present in PE, KE, or KEPE such as hydroxycinnamic acids, including caffeic acid and 3,4-dicaffeoylquinic acid, exert a direct anticancer action by inducing apoptosis via the Fas/FasL system, increasing the ratio of Bax:Bcl_2_ protein expression, and inducing the cleavage of procaspase-3 into active caspase-3^50^. Hydroxybenzoic acids, such as gallic acid and ellagic acid, induce apoptosis by elevating reactive oxygen species, disrupting matrix metalloproteinase, and activating caspase-3^9^. Mangiferin, present in mango, inhibits cell proliferation and metastatic ability in breast cancer cells by inhibiting the activation of the β-catenin pathway, lowering the expression of matrix metalloproteinase-7, -9, and vimentin, and causing the reversal of the epithelial-mesenchymal transition^51,52^. Moreover, phytoestrogens in KE, PE, and KEPE, and their derivatives confer their anticancer actions through cell cycle arrest and inhibition of the activities of certain P450 isozymes, such as CYP1A1, which are involved in pro-carcinogen activation.

On the other hand, treatment with KE, PE, or KEPE after DMBA administration reduced OS, lipid peroxidation, and mammary damage resulting from DMBA and its metabolites. As shown from the present results, the MDA levels decreased, while the GSH levels and the activities of SOD, GPx, GSR, and GST were increased as compared to the DMBA group. The reduction in OS may be related to the antioxidant effects of the PE and KE contents, including different phenolic, flavonoid, and other compounds mentioned before. These compounds scavenged the superoxide anion radicals, decreased the rate of hydroxyl formation, and quenched free radicals, which led to decreased reactive oxygen species and MDA formation. Also, phenolic acids are characterized by electron donor properties, which result in neutralizing free radicals and forming stable products that terminate the radical chain reactions^9^. Likewise, KE and PE treatments could possibly modulate the antioxidant system that could decompose the peroxide, thereby offering protection against lipid peroxidation. Significant elevation of GSH in the rats treated with PE or KE after DMBA administration as compared with the DMBA group may be related to the effects of flavonoids (quercetin, kaempferol, rhamnetin, and their related conjugates) and ellagic acid in PE, which enhance the expression of γ-glutamyl cysteine synthetase, which is the rate-limiting enzyme of GSH synthesis ^9^. In addition, phytosterols in KE, such as campesterol, β-sitosterol, ∆-avenasterol, and stigmasterol, stimulate the activity of antioxidant enzymes, especially SOD and GPx^53^.

Otherwise, our results showed that the treatment with KE and PE in combination (KEPE) after DMBA administration gave a moderate result between KE and PE. Thus, we proposed that the antioxidant effect of some phenolic compounds in PE neutralizes or antagonizes the effect of those in KE. In addition, the results showed that both KE and PE contain chlorogenic acid, caffeic acid, 3,4-dicaffeoyl quinic acid, gallic acid, ellagic acid, quercetin, and mangiferin. So, the combination treatment (KEPE) led to an elevation in the concentration of these phenolics and their accumulation in the rat body, which may cause adverse effects if given for a long period of time^10,18^.

On the other hand, the administration of KE and PE, separately, to the healthy rats for 4 weeks revealed non-significant changes (an increase or decrease) in most markers (OS and apoptosis). There were no significant differences in the histopathological examination and immunohistochemical expression of caspase-3 and ER-α as compared to the C group. This means that KE and PE are nontoxic to healthy rats.

## Conclusion

Mango extracts (PE, KE, and KEPE) combated breast cancer induced by DMBA through different mechanisms. These extracts have a strong antioxidant effect, where they reduced OS (decreased MDA levels and restore antioxidant status). Also, they have an antiproliferative and proapoptotic effect, since they increased caspase-3 activity, DNAF, and caspase-3 expression. Furthermore, these extracts have an anti-estrogenic effect by lowering serum E2 levels and acting as selective estrogen receptor modulators. The advantageous effects of KE and PE may be due to the effects of their contents, especially phenolic and flavonoid compounds which are present in large quantities. Moreover, KE contains phytosterol. Mango peels and seeds must be used as a source of phenolic and flavonoid compounds (and phytosterol in the case of KE). The administration of each KE, PE, or KEPE alone has no side effects. KE, PE, and KEPE each have a therapeutic impact against DMBA-induced mammary tumors. Therefore, they may have a significant impact on the pharmacological effect.

## Materials and methods

### Chemicals and kits

5, 5'-Dithio (bis)-2-nitrobenzoic acid, D, Triton-X-100, SOD, 1, 1, 3, 3-tetra methoxy propane, GSH, oxidized glutathione (GSSG), Trisma base, and nicotinamide adenine dinucleotide phosphate hydrogen (NADPH) bovine serum albumin were obtained from Sigma Chemical Company (St. Louis, MO, USA). Perchloric acid, P-nitro benzyl chloride, and diethylenetriamine penta acetic acid were purchased from Merck Ltd. Dimethyl sulfoxide was obtained from Fluka. Diphenylamine was purchased from Avocad. Enzyme-linked immunosorbent assay (ELISA) kits for the determination of caspase-3 and E2 were obtained from Glory Science Company (Del Rio, USA) and Calbiotech Company (Austin, USA), respectively. All other kits and chemicals were obtained from Biodiagnostic Company (Cairo. Egypt).

### Preparation of mango extracts

Mango fruits (*Mangifera indica* Linn cv. Hagar, family Anacardiceae) were obtained from the local market in Alexandria, Egypt. Mango peel was obtained by removing the peel and edible flesh from the seed kernel manually using a Kitchen Peeler and Spoon. PE was prepared as described by^18,51^. The peels were air-dried for 2 days, then pulverized, and extracted with 80% ethanol in the ratio of 1:5 (w/v) by Sonicating for 3 days at 25 °C. The extract was filtered and concentrated till dryness using Rotary Evaporator at 40 °C. Then the concentrated extract was lyophilized to obtain a powdered extract (PE). PE was dissolved in 0.6% dimethyl sulfoxide as a safe concentration {DMSO: nontoxic below 10% (v/v) and LD_50_, oral, rat, 14,500 mg/kg}^54,55^ and stored in a dark bottle at 4 °C until used. KE was prepared as described by Abu Bakar et al**.**^52^. The kernel of mango was separated from the fruit and cut into small pieces, which were diced further and sun-dried. The dried samples were ground into a fine powder using a dry grinder. The kernel powder was extracted with absolute ethanol in the ratio of 1:5 (w/v) and then filtrated. The filtrate was concentrated till dryness using a rotary evaporator. The concentrated extract was dissolved in 0.6% of dimethyl sulfoxide to the desired concentration^54,55^ and stored in a dark bottle at 4 °C.

### Characterization of PE and KE

#### Determination of total phenolic compounds: It was determined in PE and KE as mg gallic acid equivalents using gallic acid as standard^56^.

Polyphenolic compounds of each extract were separated using high-performance liquid chromatography (HPLC) since 20 µl of each PE or KE was carried out using an Eclipse XDB C18 (5 µm, 4.6*150 mm) column using a mobile phase consisting of 1% (v/v) formic acid in aqueous solution: acetonitrile: 2-propanol (70:22:8), pH 2.5, at 30 °C, flow rate 0.75 ml/min and ultraviolet detection at 320 nm (Agilent technologies 1200S, Germany).

#### Determination of total flavonoid content

It was determined in mg/ml of each PE and KE as quercetin equivalents using quercetin as a standard^57^.

#### Total antioxidant capacity

It was assessed in mg/ml of the PE or KE as mg ascorbic acid equivalents by the phosphomolybdenum method using ascorbic acid as standard^58^.

### Animals

#### Ethics approval

This study protocol was reviewed and approved by the Alexandria University, Institutional Animal Care and Use Committee. The Experiment was in conformity with the “Recommendations for Handling of Laboratory Animals for Biomedical Research” and complied with the Committee on Safety and Ethical Handling Regulations for Laboratory Experiments at ALEXU. Animal studies were carried out following the ARRIVE guidelines and are in accordance with the 1964 Helsinki Declaration and its later modification or comparable ethical standards^59^.

Eighty-four healthy adult female Sprague–Dawley rats (40 days old & weighing 140–150 g) were obtained from the Faculty of Medicine, Alexandria University, Egypt. All rats were examined for health status and maintained at proper environmental conditions of temperature at 25 °C, and relative humidity at approximately 50% with a 12-h light/dark photoperiod for ten days prior to handling. The animals were then housed in polypropylene cages (six animals per cage) covered with metallic grids, and given free access to a standard diet (rodent food) and tap water throughout the study. All animals were observed daily for abnormal signs.

#### Induction of mammary gland carcinoma in rats

In rats, mammary gland carcinoma was induced using a single intragastric dose of DMBA using oral gavage (80 mg dissolved in 4 ml sesame oil/Kg body mass (bm) rat^60^. After DMBA administration, the female rats were palpated weekly for mammary tumor development. Mammary tumors were measured in mm (length × width) using a caliper. Mammary tumors could be identified as ≥ 5 mm in diameter (5–9 weeks after DMBA administration).

#### Experimental design and animal groups

**Figure 8:**
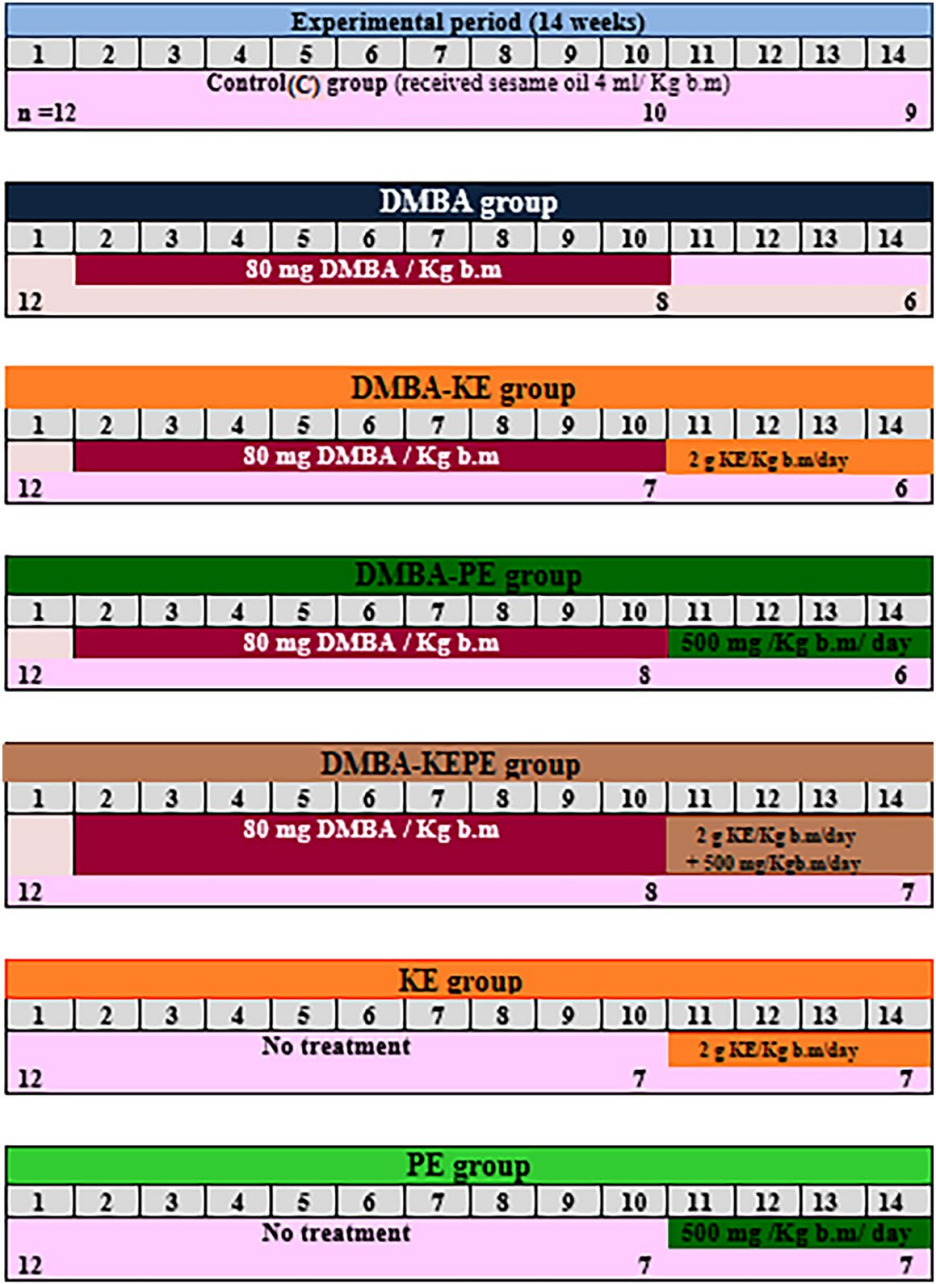
Experimental design and animal groups. The rats were divided into seven groups of twelve rats each. Sham control group (C): the rats were administered a single oral dose of 4 ml sesame oil/kg bm using oral gavage. DMBA group: the rats were administered with DMBA to induce mammary gland carcinoma. (DMBA-KE), (DMBA-PE) and (DMBA-KEPE) groups: the rats were administered with DMBA. Then after the ninth week of induction of mammary gland carcinoma, the rats were treated with KE, PE, and (KE and PE), respectively, for 4 weeks. KE and PE groups: the healthy rats were administered orally KE and PE, respectively, for 4 weeks.

After acclimatization, the rats were divided into seven groups of twelve rats each. Figure 8 shows the experimental design. Sham control group (C): the rats were administered a single oral dose of 4 ml sesame oil/kg bm using oral gavage. **DMBA group:** the rats were administered with **DMBA** to induce mammary gland carcinoma as mentioned before. (**DMBA-KE) and (DMBA-PE) groups**: the rats were administered with **DMBA** as mentioned before. After the ninth week of induction of mammary gland carcinoma, the rats in the group (**DMBA-KE)** were administered orally with 2 g of KE/kg bm/day for 4 weeks^61^, while the rats in the group **(DMBA-PE)** were administered orally with 500 mg of PE/ Kg bm/day for 4 weeks^62^. **(DMBA-KEPE) group:** the rats were administered with **DMBA**, then after the ninth week of induction of mammary gland carcinoma, rats were administered orally with both KE and PE using the same doses mentioned before. **KE and PE groups**: the healthy rats were administered orally with the same doses of KE and PE, respectively, for 4 weeks. At the end of the experimental period, feeding was stopped 12 h prior to sacrifice. Carbon dioxide gas was used to anesthetize rats for dissection. Blood samples were collected on gel-coated tubes and kept at 25 °C for 15 min to clot, centrifuged at 3000 rpm for 20 min at 25 °C to separate serum, and stored at − 80 °C until used for the determination of E_2_ level. Also, after sacrificing the rats; the mammary gland tissues were immediately removed and small portions were fixed at 10% buffered formalin for up to 18 h and afterward embedded in paraffin for histopathological and immunohistochemical examination. The remaining tissues were washed twice with ice-cold saline solution, blotted on the filter paper, dried, weighed, divided into two parts, and kept at − 80 °C until used for further analysis. The first part was used for the determination of DNAF, caspase-3, GPx, and GSR activities. The second part was homogenized in 0.1 M cold sodium phosphate buffer saline, pH 7.4 (1:5, w/v), using a Teflon glass Homogenizer at 4 °C. The homogenate was centrifuged at 7000 rpm (Hettich EBA12 Germany) for 20 min and at 4 °C and the supernatant (cytosol extract) was stored at -80 °C for determination of lipid peroxidation, protein content, GSH, GST, and SOD activities. All procedures were carried out at 4 °C.

### Biochemical assays

#### Protein content

Total protein was determined according to the method of Tsuyosh and James^63^.

#### Caspase-3 assay (EC 3.4.22.56)

Caspase-3 level was determined using a double-antibody sandwich ELISA kit^64^. The mammary gland tissues were homogenized in four volumes of phosphate-buffered saline, pH = 7.4. The homogenate was centrifuged at 7000 rpm for 20 min at 4 °C and the supernatant was used for enzyme assay. Caspase-3 activity was expressed as ng/mg protein.

#### DNAF level

The percentage of DNAF in mammary gland tissues was determined spectrophotometrically using diphenylamine^65^.

#### Status of serum E2 level

It was determined using an ELISA kit^66^ and its concentration was expressed as pg/ml.

#### Evaluation of OS

##### Lipid peroxidation level

Malondialdehyde (MDA) level, the main product of lipid peroxidation, was determined in cytosol extract as described by Ohkawa et al*.*^67^. MDA is expressed as nmol MDA/g wet tissues.

##### GSH level

It was determined in the deproteinized cytosol extract as described by Ellman^68^. GSH is expressed as mg/g wet tissues.

##### SOD (EC 1.15.1.1)

Cu–Zn–SOD activity in cytosol extract was determined as described by Marklund and Marklund^69^. The unit of enzyme activity was defined as the amount of enzyme that inhibits the rate of autoxidation of pyrogallol by 50% under standard conditions and expressed as U/mg protein/min.

##### T-GPx (EC 1.11.1.19)

MGTs were homogenized in 0.05 M potassium phosphate buffer (1:4, w/v) containing 1.15% KCl (pH 7.6). The homogenate was centrifuged at 3200 g, 4 °C for 20 min. The activity of t-GPx was determined in the supernatant at 37 °C, using cumene hydroperoxide as a substrate^70^. The specific activity is expressed as µg consumed GSH/mg protein/min.

##### GSR (EC 1.8.1.7)

MGTs were homogenized in 0.05 M potassium phosphate buffer, pH 7.2 (1:10, w/v), and centrifuged at 3200 g, 4 °C for 20 min. GSR activity was determined in the supernatant by following the oxidation of NADPH to nicotinamide adenine dinucleotide phosphate (NADP^+^) during the reduction of GSSG^71^**.** GSR activity is expressed as μmol/min/mg protein.

##### GST (EC 2.5.1.18)

GST in cytosol extract was measured spectrophotometrically, using p-nitrobenzyl chloride in 95% ethanol as substrate^72^. The specific activity of GSTs is expressed as nmol/min/mg protein.

### Histopathological examination

MGTs were fixed, processed, and embedded in paraffin wax. Sections of 5 μm in thickness were cut and stained with hematoxylin and eosin.

### Immunohistochemical detection of caspase- 3

It was carried out according to the method of Marchal et al.^73^. The slices (5 μm in thickness) were treated for blocking endogenous peroxidase activity and non-specific binding. Afterward, the sections were incubated with a monoclonal antibody against caspase-3, mouse monoclonal caspase-3 antibody [(CPP32) Ab-3 (Clone 3CSP03, NeoMarkers)] for 1 h at 37 °C in a moist chamber and then rinsed in phosphate-buffered saline. Then 3,3’-diaminobenzidine tetrachloride was added for 10 min, washed with phosphate-buffered saline, and then the immunohistochemical technique was completed with a DAB kit (DAKO) for 5 min. Nuclear counterstaining was performed with 1/2-diluted Harris hematoxylin and mounted.

### Immunohistochemical detection of ER-α

It was carried out according to the method of Katoh et al.^74^.

Immunohistochemical detection of ER-α was carried out as caspase-3 but primary antibodies of caspase-3 were replaced by the estrogen receptor (ER) antibody.

### Statistical analysis

Quantitative data were described using the mean and standard deviation for normally distributed data, while abnormally distributed data were expressed using the median, minimum, and maximum. For normally distributed data, comparison between different groups was analyzed using F-test (ANOVA) and Post Hoc test (Scheffe) for pairwise comparison. Significance test results are quoted as two-tailed probabilities. The significance of the obtained results was judged at 5%. All statistical analyses were performed using the statistical software, IBM SPSS software package, version 20.0.

## Data Availability

All data generated or analyzed during this study are included in this published article.
